# Behavioural and ERP evidence of the self-advantage of online self-relevant information

**DOI:** 10.1038/s41598-020-77538-5

**Published:** 2020-11-25

**Authors:** Gengfeng Niu, Liangshuang Yao, Fanchang Kong, Yijun Luo, Changying Duan, Xiaojun Sun, Zongkui Zhou

**Affiliations:** 1 School of Psychology, Central China Normal University, Wuhan, 430079 China; 2 Key Laboratory of Adolescent Cyberpsychology and Behavior (CCNU), Ministry of Education, Wuhan, 430079 China; 3 Center for Research On Internet Literacy and Behavior, Central China Normal University, Wuhan, 430079 China; 4 School of Psychology, Southwest University, Chongqing, 400715 China

**Keywords:** Social behaviour, Psychology

## Abstract

The present study examined whether individuals experienced the same cognitive advantage for online self-relevant information (nickname) as that experienced for information encountered in real life (real name) through two experiments at both the behavioural and neural levels (event-related potential, ERP). The results indicated that individuals showed the same cognitive advantage for nicknames and real names. At the behavioural level, a nickname was detected as quickly as the real name, and both were detected faster than a famous name; at the neural level, the P300 potential elicited by one’s nickname was similar to that elicited by one’s real name, and both the P300 amplitudes and latencies were larger and more prolonged than those elicited by other name stimuli. These results not only confirmed the cognitive advantage for one’s own nickname and indicated that this self-advantage can be extended to online information, but also indicated that the virtual self could be integrated into the self and further expanded individuals’ self-concept.

## Introduction

The self is an important conception in psychology with unique significance for individuals’ survival and development. Research on the self has always been the core issue in the field of personality and social psychology, social cognition and cognitive neuroscience^1,2^. Especially, it is of great importance for individuals to recognize oneself and distinguish ‘me’ from ‘not me’; this psychological process could not only maintain one’s sense of self but also act as the basis for various higher-order cognition (e.g., self-consciousness and the theory of mind) and social behaviours and functions (e.g., interpersonal attachment and self-control)^3,4^. Research has also suggested that individuals are equipped with extreme cognitive sensitivity and advantages (processing faster and more accurately) for various self-relevant information (one’s own name and face are most common) at both behavioural and neurological levels^5–8^.

In the current information era, the Internet, which allows various activities to be completed online, has expanded our living space and has been an indispensable part of our daily life^9,10^. In this context, the conception of a “virtual self” has been discussed, which refers to the embodiment of human characteristics within an avatar in online space^11^. Accordingly, there are new manners of self-relevant information online; for example, online nicknames and avatars, are widely used by users to represent themselves in various online settings (e.g., social networking sites, online forums, and online games). Although the online information is different from self-relevant information in words used in real life, they bear the same social functions (especially for nicknames)^11,12^. Based on these findings, this study aimed to test whether individuals show the same cognitive advantage for typical self-relevant information in online space—more specifically, for their online nicknames. This may shed new light on our understanding of the influences of the Internet on individuals, as well as the essential relationship between the virtual self and the real self.

### Cognitive advantage for self-relevant stimuli and empirical evidence

As discussed above, due to the important role of “self” and the self—representing function of self-relevant information, individuals have extreme sensitivity and a cognitive advantage for self-relevant information^5,6^. Early studies found that individuals could easily detect their own names even in a noisy environment or in the absence of attention (defined as the cocktail party effect)^13^, and information encoded with a reference to the self (associating the information with the self) could be remembered better than that encoded with a reference to others (defined as the self-reference effect)^14^. Notably, considerable studies with different experimental paradigms have indicated the attention advantage for various self-relevant stimuli, such as one’s own name, face, body, sound and handwriting^7,8,15,16^.

Accompanying the development of cognitive neuroscience in recent years, relevant research methods, mainly event-related potentials (ERPs) and functional magnetic resonance imaging (fMRI), have also been adopted to examine the neural mechanism underlying the cognitive processing of self-relevant information. fMRI studies focused on the activated area in various tasks of processing self-related information, indicating that the medial prefrontal cortex (MPFC) and related brain areas were the neural basis for self-relevant cognition, which was closely associated with self-perception, self-consciousness, and the cognitive processing of self-relevant information, such as one’s own name and face^17,18^; moreover, a recent review found that 94% of the fMRI studies on self-relevant cognitive processing found that the activation of the medial prefrontal cortex (MPFC) was related to self-related information^19^. ERP studies focused on the brain potential elicited by the cognitive processing of self-related information, as different ERP components reflect different stages of cognitive processing and cognitive engagement in the current task. The results indicated that a specific ERP component (mainly P3) was closely associated with cognitive processing for self-relevant stimuli^7,20^. For example, P3 is generally considered as the neurological index of the cognitive advantage for online self-relevant information. The amplitude and latency of P300 elicited by one’s own name or a highly self-relevant name was larger and more prolonged than that of other names, which is more prominent at the central and frontal brain^16,21,22^. All these results added to the empirical evidence for the cognitive advantage of self-relevant stimuli.

### Self-relevant information in online space

Currently, the Internet has played an increasingly important role in individuals’ daily lives with the development of information technology; to some extent, the Internet has not only greatly changed the manner of traditional lives but also expanded living spaces by constructing a virtual world^23–25^. Similar to the requirements in real life, individuals are asked to represent themselves in online space with different stimuli with text (an online nickname) or picture (a virtual avatar and a profile picture), and users adopt them in various online settings, such as instant messaging and social media to online role-playing games; such information could act as the embodiment or symbol of individuals in online space, and individuals could use them to interact with others in the Internet-based environment^24,26–28^. Given the popularity and prominence of online self-relevant information, it is of great significance to examine whether individuals would show the same cognitive advantage for typical self-relevant information in online space.

Online self-related information (e.g., nickname and virtual avatar or profile picture) is different from that in real life (e.g., name and face/body). First, individuals’ real names (usually given by the parents, and the family name is known) or faces (mainly depending on biological genetics) cannot be decided by children themselves to a large extent, whereas online self-relevant information is created or customized by users themselves, according to their own preferences. Accordingly, this information usually reflects their likes, habits and even the person they want to be (namely, their ideal and possible self)^29,30^. Second, the length of time individuals use this information is different. Self-relevant information in real life exists for a long time, especially one’s name as it exists for the whole life of a person; however, the online self-relevant information, both the nickname and the avatar, is used for a shorter time and is easier to change when compared with information in real life^28^. Third, the rules and forms are also distinct: regarding name, real name should follow certain rules and social requirements, while the nickname are unconstrained, the nickname could contain special symbols and even violate the rules of grammar; regarding face, the form of avatar could be various, and even animal or scenery picture could be adopted^31^.

However, no matter whether the self-relevant information is presented in real life or online, both types of information have similar social function—both of them are emblems of the self; namely, a publicly perceivable sign linked to images of personhood, through which individuals could present themselves, construct an identity, assume various social roles, and interact with others and the environment^12,27,32^. As discussed above, individuals were equipped with an automatic and strong processing system of self-relevant information. In particular, the self-reference effect indicated that once the information is associated with the self or processed in a self-referent or self-involved way (e.g., asking the participant to determine whether the words describe him/her), individuals would expend more cognitive resources and be processed with an advantage^3,15^. Based on these findings, this study aimed to examine the cognitive advantage for online self-relevant information, and it was hypothesized that individuals may show the same cognitive advantage for online self-relevant information as that exhibited for self-relevant information in real life.

### The current study

In addition, compared to physiological faces and avatars, real names and nicknames are more widely and frequently used in various settings both in real life and online. Thus, the real name and nickname were adopted as the offline and online self-relevant stimuli, respectively. At the same time, Chinese names consist of a family name and a first name and usually consist of three characters. Therefore, we adopted a three-character name as the study stimulus, and as the nicknames always contained more than Chinese characters, the nickname was also required to be three characters. At the same time, as behavioural and neurological studies could provide evidence from different perspectives, this study investigated this issue at the behavioural and neurological levels with two experiments: study 1 adopted a visual search paradigm to test the response time for one’s real name and nickname, and study 2 used an odd-ball paradigm to induce and test the brain activation of one’s real name and nickname. Based on the above discussion, it was specifically hypothesized that one’s nickname would be searched as fast as one’s real name, and one’s nickname would also induce the same P3 ERP potential as that of one’s real name.

This study was approved by the Bioethics Ethics Committee of Central China Normal University, and informed consent was obtained from all participants before the experiment. The experiments were performed in accordance with the Declaration of Helsinki. Specifically, the visual search task paradigm was adopted in study 1, which aimed to test the cognitive advantage for nicknames by comparing the search speed of nicknames and real names. In addition, some researchers have suggested that familiarity may be a factor influencing individuals’ cognitive advantage in information^5,15^; thus, a famous name (李克强, the current Prime Minister of China) was also included to test and exclude the influence of familiarity. Based on the behavioural findings of study 1, study 2 aimed to further test the cognitive advantage of nicknames at the neurological level with an ERP experiment. The odd-ball paradigm was adopted in this study, which examined the cognitive advantage of nicknames by comparing the brain activation elicited by a nickname, a real name, a famous name (李克强, the current Prime Minister of China), and an unfamiliar name (吴爱平).

## Results

### The results of the experiment 1

The familiarity scores for one’s real name, one’s nickname, and the famous name were 6.68, 6.25, and 5.92, respectively. This may control the influence of familiarity on the cognitive advantage. First, a one-way ANOVA was conducted to analyse the difference in the assessed familiarity of the target names, and the results showed that there was no significant difference in the familiarity of the three types of target names, F(2, 98) = 0.08, *p* > 0.05; before this, the test for homogeneity of variance (Levene’s test) was conducted, and the result [F (2, 98) = 0.83, *p* = 0.61] indicated that the names were homogeneous for familiarity.

**Figure 1:**
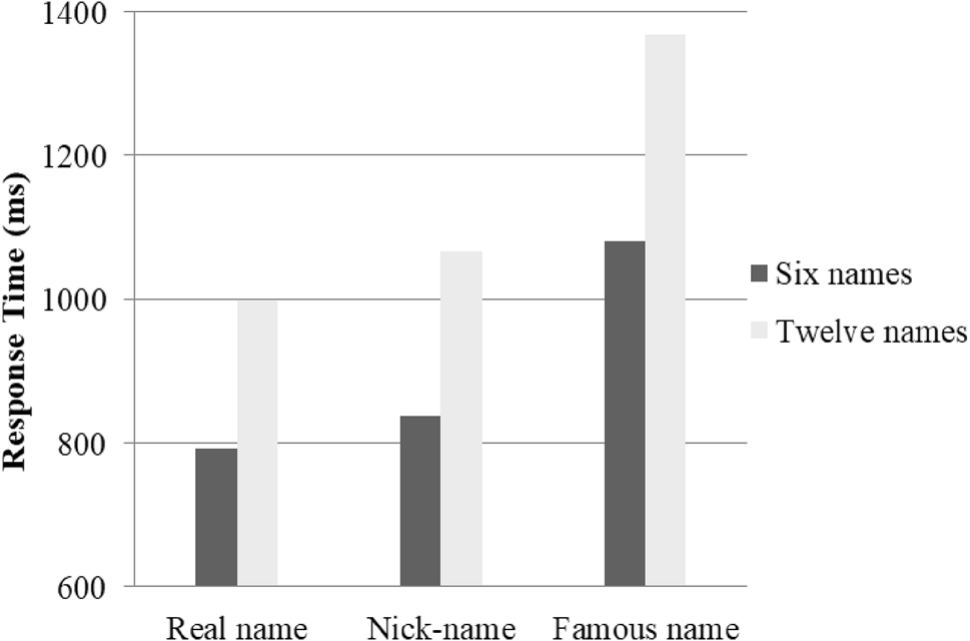
Response time for different names.

Then, a repeated 3 (the type of the target name) × 2 (the number of` the set of names) ANOVA was conducted to analyse the participants’ reaction time. The results showed significant main effects for the type of target name [F(2, 48) = 7.55, *p* < 0.001, partial *η*^2^_*p*_ = 0.64] and the number of names [F(1,48) = 12.39, *p* < 0.001, partial *η*^2^_*p*_ = 0.78]. In addition, the results also yielded a significant interaction of the type and number of names [F(2,188) = 38.48, *p* < 0.001, partial *η*^2^_*p*_ = 0.36]. Further simple main effects showed that the simple effect of the types of names was significant in both six [F (2, 48) = 30.65, *p* < 0.001] and twelve conditions [F(2, 48) = 48.33, *p* < 0.001)]. Specifically, in both conditions, there was no significant difference between the reaction time of one’s own real name and nickname (*p*_*6*_ = 0.23, *p*_12_ = 0.34), whereas the reaction time for one’s own real name and nickname were both faster than that for the famous name (both *ps* < 0.001). Namely, regardless of how many names were presented, individuals showed the same cognitive advantage for their nickname and real name (both of which were detected faster and more easily). These results indicated that individuals would also be equipped with a cognitive advantage for online self-relevant information—their online nickname. The reaction times under different conditions are presented in Fig. 1.

### The results of the experiment 2 (ERP study)

**Figure 2:**
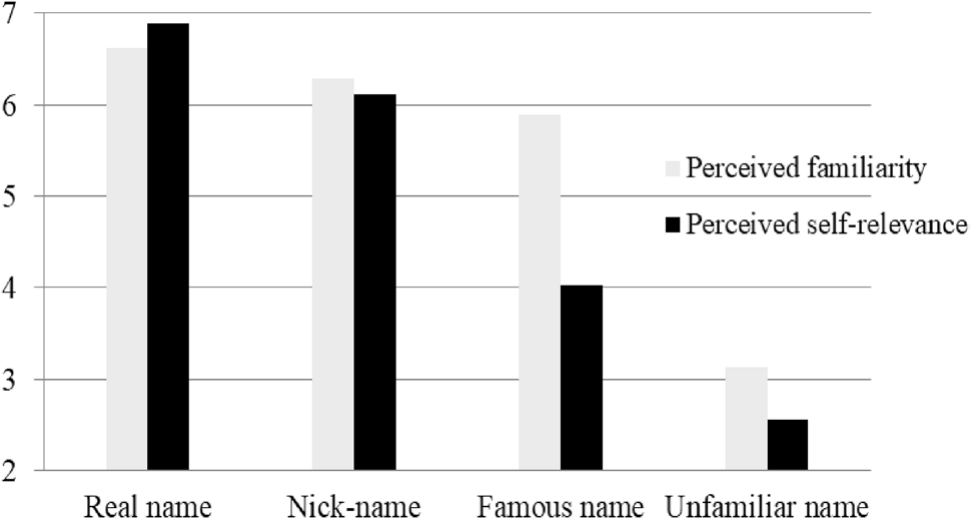
The perceived familiarity and self-relevance for different names.

Behavioural data. The familiarity scores for one’s real name, one’s nickname, the famous name and an unfamiliar name were 6.61, 6.28, 5.89 and 3.13, respectively, and the self-relevance scores for one’s real name, one’s nickname, the famous name and an unfamiliar name were 6.89, 6.11, 4.03 and 2.55, respectively (see Fig. 2). First, a one-way ANOVA found that there was a significant difference in the familiarity [*F*(3, 48) = 42.05, *p* < 0.001] and self-relevance [*F*(3, 48) = 64.78, *p* < 0.001] of the three types of target names; before this, the test for homogeneity of variance (Levene’s test) was also conducted for familiarity [F (3, 48) = 0.28, *p* = 0.12] and self-relevance [F (3, 48) = 0.95, *p* = 0.53], and the results indicated that the names were homogeneous for familiarity and self-relevance. Then, a subsequent post hoc test revealed that the familiarity scores showed no significant differences for one’s real name, one’s nickname, and the famous name, but all their scores were higher than that for the unfamiliar name (all *p*s < 0.01); for self-relevance scores, no significance existed between one’s real name and nickname, whereas the scores for both were higher than that of the famous name and unfamiliar name (both *p*s < 0.001), and the scores for the famous name were also higher than that for the unfamiliar name (*p* < 0.01).

**Figure 3:**
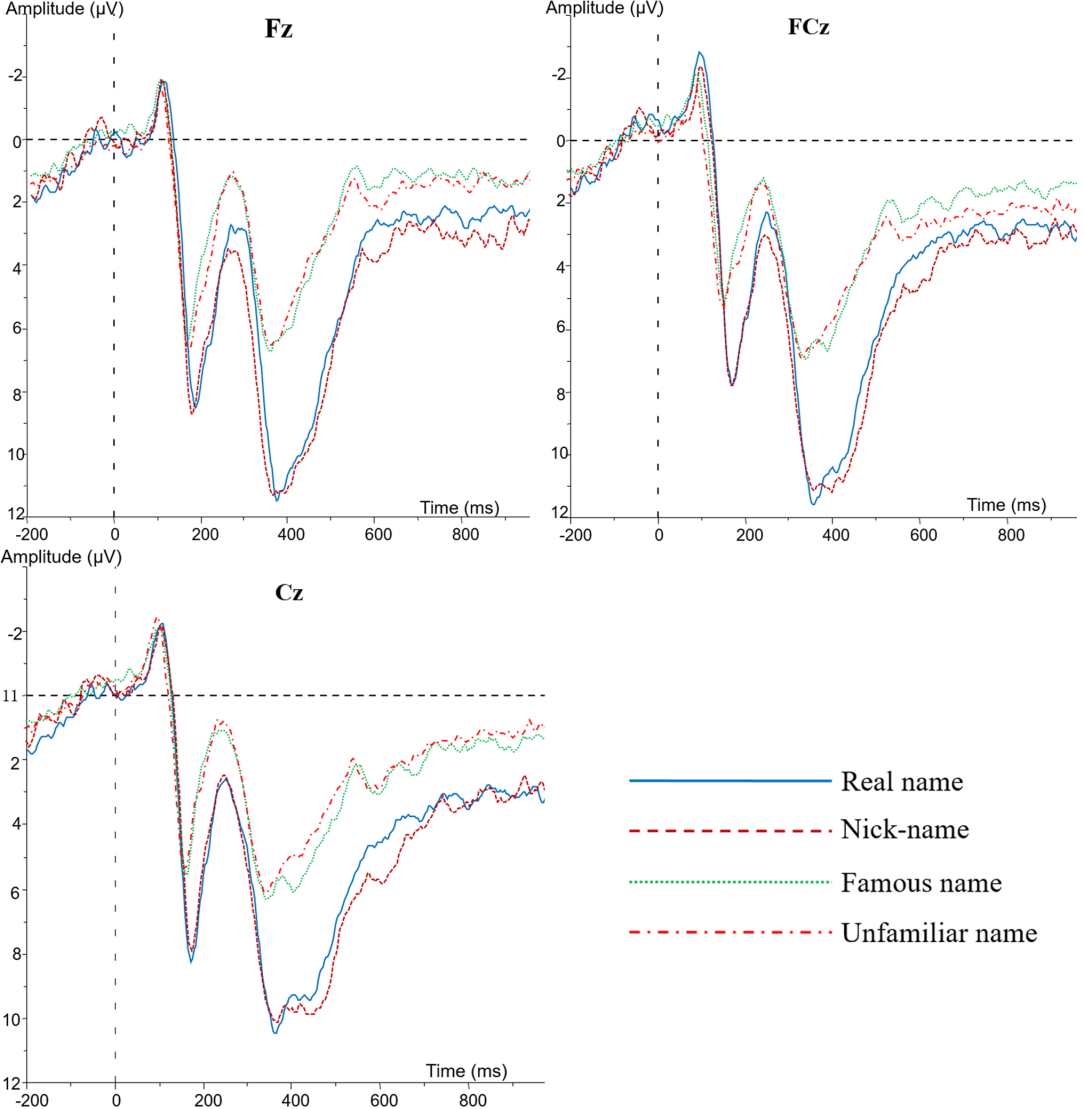
Averaged ERPs at FZ, FCZ, and CZ locations for different names.

ERP data. First, based on the peak amplitude and latency of ERP waveforms, as well as the existing literature, prominent N1 (80–120 ms), P2 (140–200 ms), N2 (260–320 ms), and P3 (320–430 ms) components were elicited during the four conditions (as presented in Fig. 3). As a researcher suggested, the early ERP component (N1) was inappropriate to analyse social cognition; thus, the P2, N2, and P3 components were used to conduct the analysis. A one-way ANOVA was conducted for the amplitudes and latencies, before which the test for homogeneity of variance (Levene’s test) was conducted, and the results indicated that the amplitudes and latencies of each component were homogeneous (both *p*s > 0.05). At the same time, as the peak values for amplitude might be more unstable for late components^34^, the value of the average amplitude was adopted in the subsequent analysis of late components (N2 and P3). In addition, the Bayesian estimation, specifically using JASP (https://jasp-stats.org/, JASP team 2017), was also conducted to further examine the hypothesis, especially the differences between real names and nicknames, with BF value as the indicator.

For the P2 component, the results of the ANOVA amplitudes demonstrated a significant effect of stimulus type [*F* (3, 48) = 10.92, *p* < 0.01, BF_10_ = 9.18]; the post hoc test revealed that both the difference between one’s real name and nickname and the difference between the famous name and an unfamiliar name were not significant (both *p*s > 0.05, the BF_10_ value of the Bayesian T-test was below 1), whereas one’s real name and one’s nickname elicited larger P2 amplitudes than the famous name and an unfamiliar name (both *p*s < 0.01, the BF_10_ value of the Bayesian T-test was above 10). Moreover, there was a significant main effect of stimulus type on P2 latencies [*F* (3, 48) = 7.15, *p* < 0.05, BF_10_ = 6.15]; specifically, one’s real name and nickname elicited more prolonged peak latencies than the famous name and an unfamiliar name (both *p*s < 0.01, the BF_10_ value of Bayesian T-test was above 6.55), whereas both the difference between one’s real name and one’s nickname and the difference between the famous name and an unfamiliar name were not significant (both *p*s > 0.05, the BF_10_ value of the Bayesian T-test was below 1).

For the N2 component, the ANOVA on the average amplitudes demonstrated a significant effect of stimulus type [*F* (3, 48) = 8.51, *p* < 0.01, BF_10_ = 7.99]; post hoc multiple-comparison further revealed that N2 amplitudes elicited by one’s real name and one’s nickname were smaller than those elicited by the famous name and an unfamiliar name (both *p*s < 0.01, the BF_10_ value of the Bayesian T-test was above 5.79), whereas both the difference between one’s real name and one’s nickname and the difference between the famous name and an unfamiliar name were also not significant (both *p*s > 0.05, the BF_10_ value of the Bayesian T-test was below 1).

Regarding the P3 component, the ANOVA on the average amplitudes revealed a significant effect of stimulus type [*F* (3, 48) = 26.54, *p* < 0.001, BF_10_ = 19.59]. The post hoc test also found that the P3 amplitudes were larger when viewing one’s real name and nickname than when viewing the famous name and an unfamiliar name (both *p*s < 0.01, the BF_10_ value of the Bayesian T-test was above 20), whereas both the difference between one’s real name and one’s nickname and the difference between the famous name and an unfamiliar name were still not significant (both *p*s > 0.05, the BF_10_ value of the Bayesian T-test was below 1). In addition, there was also a significant main effect of stimulus type on P3 latencies [*F* (3, 48) = 19.52, *p* < 0.01, BF_10_ = 14.62]. The post hoc multiple comparisons showed that one’s real name and nickname elicited more prolonged latencies than the famous name and an unfamiliar name (both *p*s < 0.01, the BF_10_ value of the Bayesian T-test was above 10); however, the difference between one’s real name and nickname and that between the famous name and an unfamiliar name were both not significant (both *p*s > 0.05, the BF_10_ value of the Bayesian T-test was below 1).

**Figure 4:**
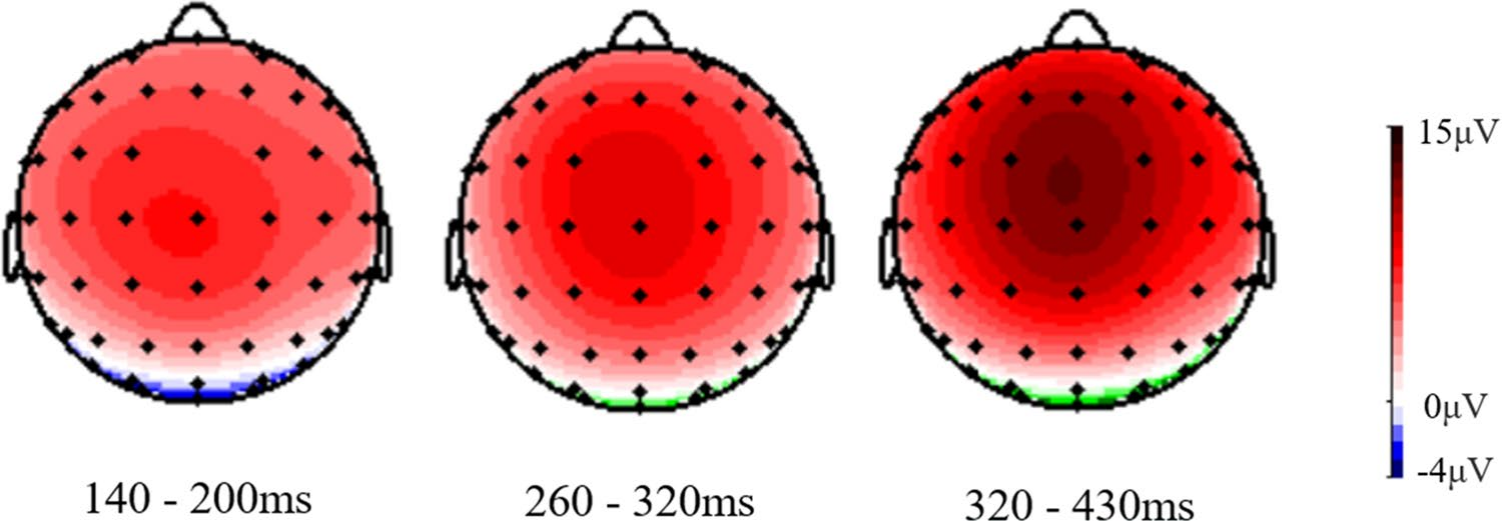
Topographical maps of the averaged ERP components.

In addition, as can been from the topographical maps presented in Fig. 4 (generated by Brain Vision Analyzer 2.0, Brain Products GmbH.), the ERP components in this study were more prominent in the central and frontal brain regions.

## Discussion

In the current information era, various self-relevant information has been created or presented online, among which one’s online nickname is one of the most common forms^24,26,29,30^. Considering the cognitive advantage for self-relevant information, as well as the same social functions of nickname and avatar with individuals’ real names and faces^12,27,28,32^, this study aimed to investigate whether individuals were equipped with the same cognitive advantage for online self-relevant information (nickname) as that in real life (real name) through two experiments at both the behavioural and neural levels. The results indicated that individuals showed the same cognitive advantage for their nicknames as they showed for their real name.

First, a behaviour experiment with a visual search task paradigm found that the response times for searching one’s real name and nickname were faster than those of the famous name at both six- and twelve-name tasks, whereas the difference in the response times between one’s real name and nickname was not significant. At the same time, consistent with previous studies^28^, the results further revealed that individuals showed the same cognitive advantage for online self-relevant information at the behavioural level, and this cognitive advantage was not associated with the familiarity of the information.

Then, an ERP experiment with an odd-ball paradigm was conducted to provide neurological evidence by comparing the brain activation elicited by participants’ real names and nicknames. At the same time, a famous name and an unfamiliar name were also included as the control stimuli to exclude the influences of familiarity and self-relevance. The results found that a clear P3 component was elicited by all four names, and the amplitude differences across conditions were most pronounced at central–parietal and frontal sites. However, one’s real name and one’s nickname elicited larger amplitudes and more prolonged latencies than the famous name and an unfamiliar name, while the difference between one’s real name and one’s nickname, as well as the difference between the famous name and an unfamiliar name, were not significant. More prolonged latencies and larger amplitudes of P3 indicated prolonged cognitive processing, as well as stronger motivational response and more cognitive engagement^20,21^. These findings suggested similar cognitive processing mechanisms between nicknames and real names, especially the P3 results; P3 is generally considered the neurological index of the cognitive advantage for online self-relevant information^16,21,22^. At the same time, the results also found that there were no significant differences among the familiarity of one’s real name, one’s nickname, and the famous name, but all these scores were higher than that for an unfamiliar name; at the same time, both the difference in the self-relevance between one’s real name and one’s nickname and the difference between the famous name and an unfamiliar name were not significant, but the self-relevance of one’s real name and one’s nickname was higher than that for the famous name and for an unfamiliar name. These results not only further confirmed the same cognitive advantage for online self-relevant information with neurological evidence but also indicated that this cognitive advantage is associated with self-relevance rather than the familiarity of the names.

Due to the close relation to self and social significance of self, many studies have indicated that individuals show cognitive advantage for various manners of self-relevant information. In particular, individuals were equipped with an automatic and strong processing system for self-relevant information, information associated with the self would be paid more cognitive resources and processed with an advantage^3,15^. At the same time, the self is theoretically defined as an abstract representation of past experience with personal data^33^, and a considerable amount of research has concluded that meaningful social groups and identity (e.g., community, school, hometown, and work), materials or objects (e.g., personal belongings and the brand one loves) and persons could become integrated into one’s representation of the self^34–38^. As the main representation of online self-relevant information, one’s online nickname can be considered a process of self-construction, which also assumes the same social function as one’s real name – individuals use the nickname to represent themselves and to interact with others and the environment in online space^12,28^. Thus, special psychological meanings were attached to the nickname during frequent usage, making the nickname closely associated with one’s self and identity and become as psychologically salient as the real name. Therefore, individuals showed the same cognitive advantage for online self-relevant information (nickname) as that shown in real life (real name).

In addition to the P3 component, other findings in experiments also provided indirect evidence for the cognitive advantage. In the odd-ball paradigm, the P2 component usually reflects automatic attention (especially attentional alertness), and it has been found that stimuli with higher emotional valence activate a more significant P2 component; the N2 component usually indicates the degree of voluntary attention motivated by higher-order cognitive functions^21,26,27^. Due to the close relation of the nickname with self and personal salience, cognitive processing of nickname was involved with strong emotional and motivational responses; thus, it could activate similar P2 and N2 components as those elicited by real names, which was significantly different from those elicited by famous and unfamiliar names. At the same time, the N1 component is associated with the physical characteristics of the stimulus^39^, and all the target stimuli comprised three characters with similar physical characteristics (e.g., length and structure). Thus, there was no significant difference in the N1 component among the names. At the same time, the results also found that the ERP components in this study were more prominent in the central and frontal brain regions, which is also consistent with previous findings on cognitive processing for self-relevant stimuli^16,19,21^.

In summary, based on previous study findings^28^, this study not only confirmed the cognitive advantage for one’s own nickname but also indicated that this self-advantage can be extended to online space. At the same time, self-expansion is an important aspect of social motivation, which refers to the phenomenon that individuals are fundamentally motivated to enhance their ability to achieve goals by the acquisition of new abilities, perspectives, identities, and resources^40,42^. Relevant studies indicated that individuals could achieve self-expansion through integrating other persons and objects into their self-concept^36,37^, and the results on self-relevant cognitive advantage also provide empirical evidence for self-expansion. For example, Aron et al. (1991) revealed a similar cognition mechanism for the self and for a partner^42^; in Chinese collectivism culture, the self-reference effect is not significant when compared with a participant’s mother^43^, and relevant fMRI and ERP studies also found that the information related to one’s mother would elicit similar brain activation patterns as those elicited for the self^44,45^. It is suggested that the self is theoretically defined as an abstract representation of past experience^33^, which could further indicate that the virtual self could be integrated into the self and expand individuals’ self-concept. However, more studies are needed to address this issue; these studies could provide an integrated perspective on the influences of Internet use and the essential relationship between online and offline life.

In addition, some limitations for this study should also be acknowledged. First, various types of self-related information are online aside from online nicknames, such as avatars, and this information should also be examined in future studies. Second, personal nicknames also exist in real life (usually created by one’s families or classmates); this concept was not included in the current experimental design. These nicknames (especially those favoured by individuals) should be considered in future studies to further examine this issue by comparing online nicknames and personal nicknames in real life.

## Methods

### Study 1: the cognitive advantage for nicknames at the behavioural level

#### Participants

Fifty undergraduate students (21 boys and 29 girls, *M*_age_ = 19.54 ± 1.41 years) were recruited to participate in this experiment, whose real names and nicknames (used in social media, instant messaging, and massively multiplayer online games) each had three Chinese characters. The nicknames included in the study were currently used and had been used for more than three years (mean usage time was 3.59 ± 1.76 years, and the average time participants used the nickname in online activities was 10.25 ± 3.65 h). The participants could obtain a pay of 15 RMB (approximately $2) for their participation after completing the experiment.

#### Stimuli

The stimuli in this experiment were four kinds of names with three Chinese characters – the participant’s real name and nickname, a famous name (Keqiang Li, 李克强), and one hundred three-character names acting as the distracting or background stimuli (half of them were female names).

One hundred names were generated through the following procedure: first, to guarantee the ecological validity of the names, 150 typical male names and 150 typical female names were gathered through an open-ended questionnaire online. Second, 15 undergraduate students were recruited to determine these names using three questions—“(1) Is the name masculine (for male names) or feminine (for female names)? (2) Is the name familiar to you? (3) Is the name obscure to you?”. If more than half of the participants provided a negative response for question 1 or indicated an affirmative response for question 2 or 3 to one name, the name was removed. After this process, 86 male names and 95 female names remained. Third, 30 undergraduate students were recruited to rate the familiarity and meaning of the remaining names on a 7-point scale, and the 50 male names and 50 female names that received scores in the middle were selected at last, and statistical analysis showed that there was no significant difference in the scores of male and female names on the perceived familiarity and meaning (both *ps* > 0.05). In addition, to eliminate potential interference, participants were asked to cross out the names they found too prominent (e.g., similar to the names of their family and friends or that were too strange to them); these names were excluded from the set of stimuli for the participants.

#### Experimental procedure

The visual search task paradigm used by previous studies was adopted in this study^28,46^. In this paradigm, the participants were asked to search for a specified name (termed the target name) from a set of names and to respond as quickly and accurately as possible. A 3 (the type of the target name: real name, nickname, and a famous name) × 2 (the number in a set of names: 6 VS. 12) within-subject design was employed. The experiment consisted of 16 blocks, each block included 48 trials—only half of which contained the target name (namely, 24 trials in a block contained the target name), and the participant was asked to indicate whether the target was presented in each trial by pressing the specific key (“F” for YES, “J” for NO).

The experimental procedure also followed relevant research^28^—in each trial, an instruction for the participants was first presented, followed by a 500 ms fixation point and a 500 ms blank screen; then, six or twelve names evenly distributed around the fixation point were presented, which remained on the screen until the participant gave the required response; then, the next trial was presented after a blank screen was shown for 1000 ms. In the experiment, the probability in which the target name was shown in one of the six or twelve positions was equal, and the other names were selected randomly from the 100 distracting names. Before the formal experiment, all participants were asked to go through a practice experiment with 20 trials to ensure that they were familiar with the procedure. In addition, the stimuli were presented, and participants’ responses (especially the reaction time—from the first showing of the set of names to the response of participants as required) were recorded automatically through the E-prime 2.0 software. Trials with no response, a false response or extremely long or short reaction times (reaction time above/below 3 standard deviations from the mean) were deleted, the number of which was less than 1% of the total trials.

Finally, after completing the experiment, participants were asked to assess their familiarity with the target names on a 7-point scale (1 “not familiar at all” to 7 “extremely familiar”).

### Study 2: the cognitive advantage for nicknames with ERP evidence

#### Participants

Seventeen undergraduate students (8 boys and 9 girls, *M*_age_ = 20.15 ± 1.27 years) were recruited to participate in this experiment. Similar to the requirement in study 1, both the real names and nicknames of the participants comprised three Chinese characters; in addition, the nicknames were currently being used and had been used for more than three years (mean usage time was 3.77 ± 1.74 years, and the average use time was 10.87 ± 3.15 h). After completing the experiment, the participants could obtain 50 RMB (approximately $7) for their participation.

#### Stimuli

Six categories of stimuli were used in this study: four names and two circles (a large circle and a small circle). In addition to the thee names (participant’s real name and nickname, and the famous name—Keqiang Li) used in study 1, an unfamiliar name (Aiping Wu, 吴爱平) was also adopted, which had been standardized and used in previous studies^8^.

#### Experimental procedure

The odd-ball paradigm used in previous research was adopted, in which the different categories of stimuli were presented for different durations^21^—a large circle was presented 840 times (70%), and a small circle was presented 72 times (6%); at the same time, each category of names was also presented 72 times (6%). The entire experiment was divided into six blocks, and the sequence of stimuli was randomized for each subject to eliminate its potential influences on the results.

The experiment was conducted according to the procedures of relevant research^16,21^. Participants were seated in an electrically shielded dark environment approximately 120 cm from a computer screen. Each trial started with a 500 ms presentation of a small white cross on a black screen, followed by a blank screen with a duration ranging from 300 to 800 ms; then, one of the stimuli was presented, and the participant was asked to observe the stimulus carefully and indicate whether the stimulus was a small circle (no response for all other stimuli). The stimuli would remain visible for 1000 ms or disappear as soon as the participant gave the response, which was followed by a blank screen lasting for 1000 ms. Between the blocks, participants could take a rest as needed. Before the formal experiment, all the participants were asked to go through a practice experiment with 25 trials to familiarize them with the experiment task, and the experiment procedure was conducted automatically through E-prime 2.0. In addition, participants were asked to assess their self-relevance (1 ‘not self-related at all’ to 7 ‘extremely self-related’) and their familiarity with the names on a 7-point scale (1 “not familiar at all”- 7 “very familiar”).

#### EEG recordings and analysis

Electroencephalography (EEG) data were recorded from 64 scalp sites, with Ag–AgCl electrodes were mounted on an elastic cap (ActiCAP, Munich, Germany) and positioned according to the extended 10–20 system. The EEG acquisition parameter was set according to the common principles of relevant research^16,21^—Electrode impedance was kept below 5 kΩ, electrode impedance was maintained below 5 kΩ, EEG and EOG activity were amplified with a DC-100 Hz bandpass, and the sampling rate was 500 Hz. At the same time, EEG data were corrected to a 200 ms baseline prior to the onset of the target, and artefact-free EEG segments to trials with correct responses were averaged for each name condition separately. ERP analysis was conducted off-line with Brain Vision Analyzer 2.0 (Brain Products GmbH., Germany)—all EEG data were filtered offline with a bandpass of 0.1–30 Hz; trials with EOG artefacts (mean EOG voltage exceeding ± 80 V), amplifier clipping artefacts, or peak-to-peak deflection exceeding ± 80 V were excluded, and the number of valid trials for each name was more than 60. ERP waveforms were time-locked to the onset of stimuli, and according to relevant studies^8,21^ and the scalp topographies (see Fig. 4), different electrode sites were selected to conduct statistical analysis for specific ERP components. For P2, five electrode sites (FCZ, C3, CZ, C4, and CPZ) were adopted; for N2, seven electrode sites (FZ, FC3, FCZ, FC4, C3, CZ, C4) were adopted; and for P3, eight electrode sites (AFZ, F3, FZ, F4, FC3, FCZ, FC4, CZ) were adopted.

## Data Availability

The data that support the findings of this study are available from Key Laboratory of Adolescent Cyberpsychology and Behavior (CCNU). Restrictions apply to the availability of these data, which were used under license for this study. Data are available from the corresponding authors with the permission of Key Laboratory of Adolescent Cyberpsychology and Behavior (CCNU).
